# Recurrent Barrett's esophagus and adenocarcinoma after esophagectomy

**DOI:** 10.1186/1471-230X-4-18

**Published:** 2004-08-25

**Authors:** Herbert C Wolfsen, Lois L Hemminger, Kenneth R DeVault

**Affiliations:** 1 Division of Gastroenterology and Hepatology, Mayo Clinic, Jacksonville, Florida, USA

## Abstract

**Background:**

Esophagectomy is considered the gold standard for the treatment of high-grade dysplasia in Barrett's esophagus (BE) and for noninvasive adenocarcinoma (ACA) of the distal esophagus. If all of the metaplastic epithelium is removed, the patient is considered "cured". Despite this, BE has been reported in patients who have previously undergone esophagectomy. It is often debated whether this is "new" BE or the result of an esophagectomy that did not include a sufficiently proximal margin. Our aim was to determine if BE recurred in esophagectomy patients where the entire segment of BE had been removed.

**Methods:**

Records were searched for patients who had undergone esophagectomy for cure at our institution. Records were reviewed for surgical, endoscopic, and histopathologic findings. The patients in whom we have endoscopic follow-up are the subjects of this report.

**Results:**

Since 1995, 45 patients have undergone esophagectomy for cure for Barrett's dysplasia or localized ACA. Thirty-six of these 45 patients underwent endoscopy after surgery including 8/45 patients (18%) with recurrent Barrett's metaplasia or neoplasia after curative resection.

**Conclusion:**

Recurrent Barrett's esophagus or adenocarcinoma after esophagectomy was common in our patients who underwent at least one endoscopy after surgery. This appears to represent the development of metachronous disease after complete resection of esophageal disease. Half of these patients have required subsequent treatment thus far, either repeat surgery or photodynamic therapy. These results support the use of endoscopic surveillance in patients who have undergone "curative" esophagectomy for Barrett's dysplasia or localized cancer.

## Background

The incidence of esophageal adenocarcinoma has increased more rapidly than any other form of cancer since the 1970s and now represents the majority of esophageal neoplasms in the West [1]. Barrett's esophagus is the replacement of native squamous mucosa by specialized intestinal metaplasia and is known to be the major risk factor for the development of adenocarcinoma via the metaplasia-dysplasia-neoplasia sequence [2]. Other risk factors for the development of esophageal adenocarcinoma include a lengthy and severe history of gastroesophageal reflux disease (GERD), increased body mass index, male gender and Caucasian race [3-5]. Recent studies, however, have detected Barrett's esophagus in nearly equal numbers of older white men whether or not they reported GERD symptoms [6,7]. Recommendations, therefore, regarding screening and surveillance of patients at risk for esophageal adenocarcinoma are controversial [2,8].

Detection of dysplastic Barrett's esophagus or mucosal adenocarcinoma is important because it allows the opportunity to intervene prior to the development of invasive neoplasia. Unfortunately, no medical or surgical GERD treatment has been consistently and convincingly demonstrated to prevent the development of adenocarcinoma [5]. Traditionally, high grade Barrett's dysplasia and mucosal adenocarcinoma are treated by surgical resection of the esophagus [9]. As endoscopic methods of screening and surveillance have become more widely applied, curative esophagectomy has produced increasing numbers of long-term survivors [10,11]. Coincident with this trend has been the appearance of several reports describing the development of metachronous esophageal adenocarcinoma after undergoing curative esophagectomy for the primary tumor [12,13]. These reports raise questions regarding the role of endoscopic surveillance in these patients. The aim of the current study was to evaluate patients who had undergone complete, presumably curative, esophageal resection for early Barrett's adenocarcinoma or high grade dysplasia in order to determine how frequently, and over what time period, they developed recurrent Barrett's esophagus or adenocarcinoma.

## Methods

After approval by the Mayo Foundation's Institutional Review Board for Research, the electronic medical records of Mayo Clinic patients in Jacksonville, Florida, were searched to find all patients who had undergone esophagectomy for cure at the Mayo Clinic surgical facility, St. Luke's Hospital, Jacksonville, Florida, since 1995. This time period was chosen to coincide with the routine availability and clinical use of pre-operative staging with endosonography in our institution. The records of these patients were reviewed for pre-operative and post-operative staging results including computed tomography and endosonography studies. In addition, endoscopic, surgical studies and histopathological studies were studied. Specifically, the surgical specimens were reviewed to ensure that the esophagectomy specimen, including lymph node sampling, was adequate and the proximal margin was completely free of Barrett's metaplasia, dysplasia or carcinoma. The patients, in whom we have at least one follow-up endoscopy study, with biopsies obtained for histologic confirmation of mucosal disease, are the subjects of this report. Esophageal disease was staged according to the Tumor-Lymph node-Metastasis (TNM) criteria [14].

## Results

Since 1995, 45 patients have undergone esophagectomy for Barrett's dysplasia or localized adenocarcinoma with curative intent in our institution. At operation, none of these patients were found to have extension of malignant disease to paraesophageal lymph nodes and all esophageal glandular mucosa was resected with only normal squamous mucosa remaining at the proximal surgical margin. Subsequently, 36 of these patients (80%) have undergone endoscopy after surgery including 8/45 patients (18%) who were found to have recurrent Barrett's glandular mucosa after curative resection and are described in the table.

Open transthoracic esophagectomy (Ivor Lewis procedure) with pyloroplasty was performed in most patients (39/45) including the patients diagnosed with recurrent Barrett's disease. Five different surgeons performed these operations. Most patients had evidence of gastric stasis (retained food) at endoscopy (30/36; 83%). Anastomotic dilation was performed at endoscopy in 16/36 patients (range of dilation procedures 1–10; median 3). It is possible that patients with anastomotic strictures may be at increased risk of recurrent Barrett's esophagus because of worse reflux although their swallowing symptoms may, alternatively, be related to other factors such as anastomotic ischemia or surgical sutures. Patients frequently used aspirin (42%) or COX-2 specific non-steroidal anti-inflammatory agents (25%). Twice-daily proton pump inhibitors were routinely prescribed for these patients although patient compliance is difficult to assess because of high drug costs and limited symptomatic improvement. While the small number of patients limits our analysis, these factors were found to occur in a proportional number of patients with Barrett's disease and no clear trends could be identified.

Five of these patients have been diagnosed with Barrett's metaplasia or low-grade dysplasia have been followed for more than 12 months in surveillance endoscopy programs monitoring the stability of the glandular epithelium. Two of these 5 patients have been found to have short segment Barrett's metaplasia with lengths of 10 mm and 15 mm, after complete esophageal resection for Barrett's high-grade dysplasia in a 72-year-old man and Barrett's adenocarcinoma T_2_N_0_M_0 _in a 78-year-old man. This metaplastic glandular epithelium was detected at a follow up of 90 months and 17 months, respectively. In the other 3 patients, short segment Barrett's low-grade dysplasia has been found in lengths between 10–25 mm after complete resection of the esophagus for Barrett's high-grade dysplasia (1 patient) and Barrett's T_3_N_0_M_0 _carcinoma (2 patients). This recurrent Barrett's glandular dysplasia was detected at a follow up of 42–47 months. Erosive esophagitis was also noted in 4 of 5 patients indicating uncontrolled reflux disease. Subsequently all patients have been treated with high doses of proton pump inhibitors (such as esomeprazole 80 mg twice a day or 40 mg three or four times per day) in an attempt to maximally control reflux of acid and digestive juices from the stomach into the cervical esophagus.

Three other patients developed recurrent Barrett's disease after curative resection of esophageal T_2 _or T_3_N_0_M_0 _adenocarcinoma. These patients varied in age from 58–80 years of age. These patients were found to have more severe erosive esophagitis suggesting worse acid reflux and mucosal injury compared to the non-carcinoma recurrent Barrett's patients. Recurrent Barrett's multi-focal high-grade dysplasia, over a 10 mm segment length was detected 88 months after esophagectomy in one patient and was successfully ablated with porfimer sodium photodynamic therapy using the methods described elsewhere [15]. High-grade dysplasia with features of invasive adenocarcinoma was noted in a 20 mm Barrett's segment diagnosed 18 months after esophageal resection in another patient. Endosonography detected focal mucosal expansion and the Barrett's mucosal adenocarcinoma T_1_N_0_M_0 _was confirmed at repeat esophagectomy. Finally, a diminutive polypoid mass proximal to the surgical anastomosis was found in a 58-year-old woman who had 7 months previously undergone esophagectomy for Barrett's mucosal adenocarcinoma. Computed tomography with contrast enhancement noted esophageal wall thickening and suspicious lymphadenopathy. Repeat resection confirmed the tumor histologic grade of T_2_N_1_M_0 _adenocarcinoma.

## Discussion

Over the past four decades, the incidence of esophageal adenocarcinoma has risen dramatically, particularly in older white men [16]. Previous studies have documented the association of esophageal cancer with the severity and duration of gastroesophageal reflux [3]. The most important risk factor for the development of esophageal adenocarcinoma, however, is the development of specialized intestinal metaplasia in the lower esophagus (Barrett's esophagus) [5]. It is recommended that patients with Barrett's mucosa undergo periodic surveillance endoscopy with systematic biopsies to detect the presence of cancerous or pre-cancerous changes (dysplasia or neoplasia) [2]. In these patients, especially, Barrett's adenocarcinoma or high-grade dysplasia is found at an early stage when it is increasingly possible to undergo esophagectomy with curative intent [17].

After undergoing esophageal resection, the native squamous mucosa of the cervical esophagus will be brought into contact with the acid-secreting mucosa of the gastric body. This reconstruction allows acid and duodenal juice to reflux from the gastric conduit to the remaining cervical esophagus. Reflux of gastric and duodenal content is an important factor in the pathogenesis of Barrett's metaplasia, dysplasia and esophageal adenocarcinoma [18-20]. It is well known that recurrent Barrett's glandular mucosa is frequently found in the cervical esophagus after esophageal resection [21-23]. Recently, Oberg et al performed endoscopy, esophageal manometry and ambulatory pH studies in 32 patients who had undergone esophagectomy for a variety of diagnoses including 16 patients (50%) with adenocarcinoma associated with Barrett's metaplasia [24]. These studies were performed between 3–10 years after the surgery and most of the patients (70%) were receiving at least once daily proton pump inhibitor therapy for chronic reflux symptoms. At endoscopy, Barrett's glandular mucosa was histologically documented in 15 patients (47%) ranging in segment lengths from 0.5–4.0 cm. Importantly, recurrent Barrett's glandular mucosa was significantly more likely to occur in patients with a preoperative diagnosis of Barrett's epithelium suggesting that the esophageal mucosa of these patients may be pathogenetically predisposed to develop metaplastic changes in response to gastroesophageal reflux.

In the study of Oberg et al, despite the use of potent acid-suppressing medications, severe esophageal acid exposure was noted in most patients. Patients with recurrent Barrett's epithelium were found to have significantly more severe acid exposure that occurred predominantly in the supine position [25]. There was also a direct correlation between the length of the metaplastic segment and the severity of acid reflux. Similar findings were reported by da Rocha, et al., who studied 48 patients after esophagectomy where 4 of these patents (8%) were found to have pathological changes of Barrett's metaplasia in the cervical esophageal stump [26]. In both of these studies, the authors concluded that the finding of recurrent Barrett's esophagus was related to reflux esophagitis that resulted from the action of acid-peptic and biliary secretions. These studies, however, did not detect dysplasia or neoplasia and did not address the issue of metachronous cancers and the role of endoscopic surveillance for these patients.

Murata and colleagues recently reported the diagnosis of metachronous squamous cell carcinomas in five of 253 patients (2%) who had undergone esophagectomy for thoracic esophageal squamous cell carcinoma more than two years previously. These superficial carcinomas (T_is _or T_1_) were detected at surveillance endoscopy and were treated with endoscopic laser ablation, mucosal resection or surgical resection. While squamous cell carcinomas are not related to gastroesophageal reflux, this paper also suggests that esophageal cancer patients (squamous or adenocarcinoma) are predisposed to the development of metachronous carcinomas in the remnant cervical esophagus. This is consistent with DeMeester's experimental model of Barrett's dysplasia and adenocarcinoma occurring after complete gastrectomy with esophago-jejunostomy and reflux of bile and digestive enzymes into the cervical esophagus [27]. Also, it is likely that these patients had other important risk factors, such as tobacco and alcohol use, that predispose to both squamous and adenocarcinomas [5].

Konishi et al reported finding an adenocarcinoma in Barrett's esophagus following a total resection of the gastric remnant in a 52-year-old man who had undergone distal gastrectomy for gastric cancer nearly twenty years previously [28]. The Barrett's mucosa associated with the adenocarcinoma contained high grade dysplasia supporting the acquired theory of pathogenesis for Barrett's esophagus that suggests that reflux esophagitis after gastrectomy may result in the dysplasia-carcinoma sequence. In addition, Streitz et al retrospectively reviewed long-term survivors after esophagectomy for adenocarcinoma [13]. With a follow-up as long as 14 years, they found 4 patients who subsequently were diagnosed with esophageal adenocarcinoma. However, the time period between the development of the first and second tumors was not specified making it not possible to determine if these were recurrent tumors or new, metachronous lesions. Finally, Riben et al have reported the development of a secondary Barrett's adenocarcinoma in a patient who had 19 years previously undergone esophagectomy for a stage IIb Barrett's adenocarcinoma [12].

These studies have demonstrated that the cervical esophagus is exposed to high amounts of acid and refluxate despite the use of proton inhibitor medications and often in the absence of severe reflux symptoms. Although our group of patients has been observed for only a median of 2 years after esophagectomy, our study confirms that the development of metaplastic columnar mucosa in the cervical esophagus is a common complication related to reflux associated injury to the squamous epithelium. Further, our findings suggest that this recurrent glandular mucosa is unstable and predisposed to the development of dysplasia and invasive carcinoma, as has already developed in most of patients.

The early detection of this recurrent disease remains vitally important to preserve all possible treatment options including surveillance endoscopy follow-up, endoscopic ablation with porfimer sodium photodynamic therapy, and if necessary repeat esophagus resection surgery. Our specific recommendations include surveillance endoscopy every 6–12 months for patients who have undergone "curative" esophagectomy for Barrett's dysplasia or adenocarcinoma. In addition, we also routinely recommend indefinite use of proton pump inhibitors, regardless of symptom status, starting at twice daily dosing and increasing as necessary to control reflux symptoms and mucosal damage due to acid, bile and digestive enzymes. Whether these drug doses should be titrated based on ambulatory pH and impedance test results remains to be determined. We have generally been disappointed by prokinetic agents such as metoclopromide in improving reflux symptoms in these patients. For esophagectomy patients who develop recurrent Barrett's metaplasia we recommend the use of COX-2 inhibitors or aspirin chemoprevention to protect against the development of metachronous Barrett's carcinoma. [29,30].

## Competing interests

None declared.

## Author's contributions

All authors participated in the study design and coordination as well as case collection and review of histopathologic and endoscopic results. All authors read and approved the final manuscript.

**Table 1:** Recurrent Barrett's Disease after Esophagectomy with Curative Intent

**Pre-operative diagnosis**	**Sex**	**Age**	**F/u diagnosis**	**Time to F/U**	**Tx**
Barrett's T3 N0 ACA	F	58	Barrett's T2 N1	7 mos	Surgery
Barrett's T3 N0 ACA	F	64	Barrett's LGD	42 mos	PPI
Barrett's T3 N0 ACA	F	64	Barrett's LGD	42 mos	PPI
Barrett's HGD	M	64	Barrett's LGD	47 mos	PPI
Barrett's HGD	M	72	Barrett's Metaplasia	90 mos	PPI
Barrett's T3 N0 ACA	M	69	Barrett's T1 N0	18 mos	Surgery
Barrett's T2 N0 ACA	M	78	Barrett's metaplasia	17 mos	PPI
Barrett's T2 N0 ACA	M	80	BE+HGD	88 mos	PDT

ACA = Adenocarcinoma

HGD = High-grade dysplasia

LGD = Low-grade dysplasia

PDT = Photodynamic therapy

PPI = Proton pump inhibitor medical therapy

## Pre-publication history

The pre-publication history for this paper can be accessed here:


